# *SNAI2* and *TWIST1* in lymph node progression in early stages of NSCLC patients

**DOI:** 10.1002/cam4.1545

**Published:** 2018-05-29

**Authors:** Camille Emprou, Pauline Le Van Quyen, Jérémie Jégu, Nathalie Prim, Noëlle Weingertner, Eric Guérin, Erwan Pencreach, Michèle Legrain, Anne‐Claire Voegeli, Charlotte Leduc, Bertrand Mennecier, Pierre‐Emmanuel Falcoz, Anne Olland, Nicolas Santelmo, Elisabeth Quoix, Gilbert Massard, Dominique Guenot, Marie‐Pierre Chenard, Michèle Beau‐Faller

**Affiliations:** ^1^ Department of Pathology Hôpital de Hautepierre University Hospital of Strasbourg Strasbourg France; ^2^ Department of Public Health Nouvel Hôpital Civil University Hospital of Strasbourg Strasbourg France; ^3^ EA3430: Tumoral Progression and Micro‐environment, Translational and Epidemiological Approaches Université de Strasbourg Strasbourg France; ^4^ Department of Pneumology Nouvel Hôpital Civil University Hospital of Strasbourg Strasbourg France; ^5^ Department of Molecular Biology Oncobiology Laboratory Regional Institute of Cancer Strasbourg Hôpital de Hautepierre University Hospital of Strasbourg Strasbourg France; ^6^ Department of Thoracic Surgery Nouvel Hôpital Civil University Hospital of Strasbourg Strasbourg France

**Keywords:** epithelial‐mesenchymal transition, hypoxia, lymph node metastasis, nonsmall cell lung cancer, SNAI2, TGFβ, TWIST1

## Abstract

Lymph node metastasis is an important prognosis factor in non‐small cell lung cancer (NSCLC) patients. The aim of this study was to investigate the role of epithelial to mesenchymal transition (EMT) in lymph node progression in the early stages of NSCLC. We studied a retrospective cohort of 160 consecutive surgically treated NSCLC patients with available frozen tumor samples for expression of EMT markers (*CDH1, CTNNB1, CDH2,* and *VIMENTIN*), inducers (*TGFB1, c‐MET,* and CAIX), and transcription factors (EMT‐TF:*SNAI1, SNAI2, ZEB1, TWIST1,* and *TWIST2*). Partial EMT was more frequent in N1‐2 (N+) vs N0 patients (*P *<* *.01). *TGFB1* (*P *=* *.02) as well as *SNAI2* (*P *<* *.01) and *TWIST1* (*P *=* *.04) were the most differentially expressed genes in N+ tumors. In this group, ZEB1 was correlated with all EMT inducers and other EMT‐TFs were overexpressed depending on the inducers. CAIX was an independent prognostic factor for overall survival (IC 95% HR: 1.10‐5.14, *P *=* *.03). Partial EMT is involved in lymph node progression of NSCLC patients and depends on the TGFβ pathway. EMT‐TFs are differentially expressed depending on EMT inducers. CAIX might be a relevant prognostic marker in early stage NSCLC.

## 1 INTRODUCTION

Non‐small cell lung cancer (NSCLC) is the leading cause of cancer‐related death worldwide.^1^ Surgical resection is the best treatment for early stage NSCLC patients (I to IIIA UICC stage), which includes lymph node metastasis (N1, N2 status) or not (N0 status).^2^, ^3^ Although surgery improves overall survival (OS), local relapse or distant metastasis is frequent and leads to mortality.^4^ Lymph node status is an independent prognostic factor of NSCLC, but the mechanisms underlying progression from N0 status to N+ status remain poorly understood.^5^

During embryonic development, polarized epithelial cells can undergo morphological changes to mesenchymal cells by so‐called epithelial‐mesenchymal transition (EMT). These cells lose their epithelial features to become mesenchymal‐like cells, without cell‐to‐cell contacts.^6^ EMT also occurs in inflammatory processes and in cancer progression, in which EMT could be involved in invasion, migration, and anti‐apoptotic features as well as drug resistance.^7^ EMT is reversible, via mesenchymal‐epithelial transition (MET), which could occur in metastatic localizations.^8^

The EMT phenotype can be described analyzing epithelial markers, such as E‐Cadherin and β‐Catenin, and mesenchymal markers, such as N‐Cadherin and Vimentin.^6^, ^9^ E‐Cadherin depletion leads to nuclear relocalization of β‐Catenin, supporting EMT.^10^, ^11^ EMT effectors are also called EMT transcriptional factors (EMT‐TFs). These proteins are transcriptional repressors of epithelial markers, such as *CDH1*, which encodes the E‐cadherin protein, and/or transcriptional activators of mesenchymal markers, such as *CDH2*, which encodes the N‐Cadherin protein.^4^, ^12^, ^13^, ^14^, ^15^ EMT‐TF are represented by the zinc‐finger proteins SNAI1 (SNAIL), SNAI2 (SLUG), ZEB1, and ZEB2 as well as the basic helix‐loop‐helix factors TWIST1 and TWIST2, among others.^6^, ^14^ EMT inducers include several signaling pathways, such as TGFβ, epithelial growth factor (EGF), hepatocyte growth factor (HGF), and its receptor c‐MET, AKT‐mTOR, MAPK/ERK, NF‐ĸB, Wnt, Hedgehog, Notch, or hypoxia.^12^

In NSCLC, EMT could be an important process for development of local lymph nodes or distant metastasis.^16^, ^17^, ^18^ To our knowledge, this is the first systematic analysis of different EMT actors in early stage NSCLC depending on the lymph node status, which is the first step for lung tumor progression. The aim of this retrospective study was to describe some EMT markers, effectors, and inducers in a retrospective cohort of early stage NSCLC by comparing N0 and N+ patients.

## 2 PATIENTS AND METHODS

### 2.1 Patients and tumor tissue samples

We retrospectively reviewed all consecutive NSCLC patients treated by surgery between January 2010 and December 2012 at the University Hospital of Strasbourg, France (Figure S1). We selected patients for which sufficient formalin‐fixed paraffin‐embedded (FFPE) material and frozen sections of the tumor were available, with a tumor cellularity beyond 30% of tumor cells. Research was conducted according to the recommendations outlined in the Helsinki Declaration. Patients provided signed consent, and approval from the Institutional Review Board was obtained (Comité d'Ethique du CHU de Strasbourg, 4, rue Kirschleger, 67085 Strasbourg Cedex, France, N° 2013‐35, Pr. B. Geny, obtained October 8th, 2013). Patients were separated in two groups depending on lymph node status: the N0 group of patients without lymph node metastasis and N+ group of patients with lymph node metastasis (N1 or N2 status). Clinical data concerning age, gender, smoking history, chemotherapy, or EGFR Tyrosine Kinase Inhibitors (TKI) treatment, recurrence, and death were collected. A nonsmoker patient was defined as smoking less than 100 cigarettes in a lifetime. Pathological staging was based on the TNM (Tumor‐Node‐Metastasis) guidelines of the UICC (Union for International Cancer Control) 7th edition for the classification of lung cancer.^5^ NSCLC were classified according to the 2004 WHO classification.^19^ Adenocarcinomas (ADC) were re‐classified according to recent IASLC/ATS/ERS recommendations.^20^ Follow‐up was completed on 1 April 2015, which was defined as the deadline date.

### 2.2 Immunohistochemical analysis

Formalin‐fixed paraffin‐embedded blocks representative of the tumor were selected. Four micrometer sections were obtained from paraffin blocks. Two primary monoclonal antibodies were used: a c‐MET rabbit monoclonal antibody (SP44 clone, prediluted, Roche Tissue Diagnostics/Ventana Medical Systems, Tucson, AZ, USA) and carbonic anhydrase IX (CAIX) rabbit polyclonal antibody (Ab15086 clone, 1/1000, Cambridge, MA, USA). Immunohistochemistry (IHC) was performed using a Ventana Benchmark IHC platform. Subsequent steps were performed with the UltraView Universal DAB Detection Kit (Roche Tissue Diagnostics/Ventana Medical Systems, Tucson, AZ, USA). Two pathologists (CE, PLVQ) independently graded the staining intensity of all tumors. For each tumor specimen, the results from the two pathologists were averaged, and if the difference was over 10%, common reading and grading were performed. For c‐MET, the following intensity scores were assigned: no signal, 0; weak complete membranous signal, 1; moderate complete membranous signal (similar to normal alveolar epithelium), 2; and intense complete membranous signal, 3. For CAIX, the following intensity scores were assigned: no signal, 0; weak cytoplasmic and/or membranous signal, 1; moderate cytoplasmic and/or membranous signal, 2; and intense cytoplasmic and/or membranous signal, 3. The fraction of stained cells was determined for each intensity score. For c‐MET and CAIX, the tumor was considered positive (protein overexpression) if 50% or more tumor cells were labeled with intensity scores of 2 or 3.

### 2.3 *EGFR, KRAS, BRAF, HER2,* and *PIK3CA* mutational status and *ALK* gene rearrangement analysis

Molecular analyses were performed on FFPE tumor samples as previously described.^21^

### 2.4 Real‐time quantitative reverse transcription PCR (RT‐qPCR)

After morphological control for the presence of ≥30% tumor cells on H&E tissue sections, total RNA was extracted from frozen samples of each tumor with TRIzol reagent according to the manufacturer's protocol (Thermo Fisher Scientific, Waltham, MA, USA). Nontumoral frozen lung tissues (n = 10) were used for relative qPCR. The integrity of total RNA (RIN) was verified in all samples with an Agilent 2100 Bioanalyser (Agilent Technologies, Santa Clara, CA, USA). RNA quantification was performed by spectrophotometry (Nanodrop 2000, Thermo Fisher Scientific, Waltham, MA, USA), and complementary DNA (cDNA) was synthetized by reverse transcription (RT) from 1 μg of total RNA with the Superscript VILO cDNA Synthesis Kit (Thermo Fisher Scientific, Waltham, MA, USA). The following genes were studied: *CDH1*,* CDH2*,* CTNNB1*,* VIMENTIN*,* SNAI1*,* SNAI2*,* ZEB1*,* TWIST1*,* TWIST2*,* TGFB1*,* TGFBR1*,* TGFBR2*,* c*‐*MET*,* HIF1*α, and *HIF2*α, and normalized with the reference gene *PBGD* (QuantiTect Primer, Qiagen, Hilden, Germany). Quantitative real‐time polymerase chain reaction (qPCR) was performed using the QuantiTect SYBR Green PCR Kit (Qiagen, Hilden, Germany) in a total volume of 10 μL on a Roche LightCycler 480 Real‐Time PCR System (Roche Diagnostics, Indianapolis, IN, USA). Real‐time qPCR was performed under the following conditions: denaturation at 94°C for 15 seconds, annealing at 55°C for 30 seconds and extension at 72°C for 30 seconds. The experiments were performed in triplicate. The relative levels of gene expression were represented as ΔCt = Ct_target gene_ − Ct_reference gene_, and the relative ratios of gene expression between NSCLC tissues and a pool of nontumorous lung tissues were calculated by the 2^−ΔΔCt^ method. For *CDH1* and *CTNNB1*, gene expression was considered lost when the relative ratio values were lower than 0.5. For all other genes, overexpression was defined by a relative ratio above 2. For EMT phenotype analysis, partial EMT was defined by normal E‐Cadherin expression with N‐Cadherin overexpression, while total EMT was defined by E‐Cadherin loss of expression combined with N‐Cadherin overexpression.

### 2.5 Statistical analysis

The association of the clinical and pathological characteristics with the IHC and RT‐qPCR results was analyzed using the chi‐square test and Fisher's exact test. Overall survival (OS) was defined as the period of time from the date of surgery to the date of death or defined deadline date (1 April 2015). Relapse‐free survival (RFS) was defined as the period of time from the date of surgery to the date of relapse or defined deadline date. The Kaplan‐Meier method was used to calculate RFS or OS, and the survival curves were compared using the Log‐rank test. Univariate and multivariate analyses were performed using Cox proportional hazards models. All factors with a value of *P *≤* *.05 in univariate analysis were included in the multivariate analyses. In the analyses, *P *≤* *.05 was considered statistically significant. Statistical analyses were performed using the IBM SPSS Statistics 20.0 software for windows (IBM Corp, Armonk, NY, USA).

## 3 RESULTS

### 3.1 Clinical, pathological, and molecular characteristics of patients

There were 138 smokers (86%) and 18 (11%) never smokers (Table 1). Histological analysis reported 116 (72%) cases of adenocarcinomas (ADC), 38 (24%) cases of squamous cell carcinomas (SCC), and six (4%) cases of other types of carcinomas. Lymph node metastasis (N+) was present in 80 (50%) patients, with N1 and N2 status in 39% and 41% cases, respectively. Molecular analysis showed 15 (9%) tumors with *EGFR* mutations (four deletions, eight L858R mutations, and four other *EGFR* mutations) and 51 (32%) tumors with *KRAS* mutations (40 codon 12 mutations and 11 codon 13 mutations); the other mutations were rare (two *BRAF* V600E mutations, one *HER2* insertion, and four *PIK3CA* mutations). No *ALK* rearrangement was found. When comparing the N0 group with the N+ group, more SCC cases were present in the N+ group (n = 26 in N+ vs n = 12 in N0, respectively, *P *=* *.02). N+ patients received more frequently neoadjuvant chemotherapy (14 vs 6 in N+ and N0, respectively, *P *=* *.05) and EGFR TKI treatment (15 vs 6 in N+ and N0, respectively, *P *=* *.04).

**Table 1:** Clinical, pathological, and molecular characteristics of patients

	All patients	N0	N+	*P* value
Total N (%)	160 (100)	80 (50)	80 (50)	
Clinical features				
Age at diagnosis				
≤60 y	66 (41)	31 (39)	35 (44)	NS
>60 y	94 (59)	49 (61)	45 (56)	
Gender				
Male	109 (68)	55 (69)	54 (68)	NS
Female	51 (32)	25 (31)	26 (32)	
Smocking history				
Never smoked	18 (11)	9 (11)	9 (11)	NS
Ex‐smoker	62 (39)	24 (30)	38 (47)	
Current smoker	76 (47)	45 (56)	31 (39)	
Unknown	4 (3)	2 (3)	2 (3)	
Neoadjuvant chemotherapy				
No	140 (87)	74 (93)	66 (82)	.05
Yes	20 (13)	6 (7)	14 (18)	
TKI treatment				
No	128 (80)	67 (84)	61 (76)	.04
Yes	21 (13)	6 (7)	15 (19)	
Unknown	11 (7)	7 (9)	4 (5)	
Pathological features				
Histology				
Adenocarcinomas	116 (72)	64 (80)	52 (64)	.02
Solid predominant	39 (24)	19 (24)	20 (25)	
Acinar predominant	48 (30)	22 (28)	26 (33)	
Papillary predominant	6 (4)	4 (5)	2 (2)	
Micropapillary predominant	9 (6)	7 (9)	2 (2)	
Lepidic predominant	10 (6)	8 (10)	2 (2)	
Mucinous predominant	2 (1)	2 (2)	0 (0)	
Colloid predominant	2 (1)	2 (2)	0 (0)	
Squamous cell carcinomas	38 (24)	12 (15)	26 (33)	
Others^a^	6 (4)	4 (5)	2 (3)	
pT‐Stage				
1	39 (24)	25 (31)	14 (18)	NS
2	67 (42)	34 (42)	33 (41)	
3	43 (27)	15 (19)	28 (35)	
4	11 (7)	6 (8)	5 (6)	
Thoracic UICC Stage				
IA	25 (16)	25 (31)	0 (0)	<.001
IB	28 (17)	28 (35)	0 (0)	
IIA	26 (16)	6 (7)	20 (25)	
IIB	16 (10)	15 (19)	1 (1)	
IIIA	62 (39)	6 (8)	56 (70)	
IIIB	3 (2)	0 (0)	3 (4)	
Molecular features				
Mutation status				
EGFR mutation	15 (9)	8 (9)	7 (9)	NS
KRAS mutation	51 (32)	28 (35)	23 (29)	
BRAF mutation	2 (1)	0 (0)	2 (2)	
HER2 mutation	1 (1)	0 (0)	1 (1)	
PIK3CA mutation	4 (3)	5 (5)	1 (1)	
ALK rearrangement	0 (0)	0 (0)	0 (0)	
Wild type	87 (54)	41 (51)	46 (58)	

a Two adenosquamous carcinomas and five nonsmall cell carcinomas with neuroendocrine features.

N0 = Patients with lymph node tumor status N0; N+ = Patients with lymph node tumor status N1 or N2; TKI = EGFR Tyrosine kinase inhibitor; NS = Not significant; *P*‐value <.05 statistically significant.

### 3.2 Expression of EMT markers and effectors according to lymph node status

Most of the tumors presented epithelial marker expression; 132 (92%) tumors presented *CDH1* (E‐Cadherin) expression, and 131 (92%) tumors presented *CTNNB1* (β‐Catenin) expression (Table 2). *CDH1* and *CTNNB1* expression were not different in the N0 compared to the N+ group of tumors. Mesenchymal EMT marker analyses showed that 71 (50%) tumors presented *CDH2* (N‐Cadherin) overexpression and 18 (13%) tumors presented *VIMENTIN* overexpression. *CDH2* overexpression was significantly more frequent in the N+ group compared to the N0 group (n = 43, 60% vs n = 28, 39%, *P *=* *.01), and similar results were found for *VIMENTIN* overexpression (n = 13, 18% in the N+ group vs n = 5, 7% in the N0 group, *P *=* *.04). Partial EMT was observed in half of the tumors (n = 70, 49%) and only one tumor presented a total EMT phenotype (in the N+ group—data not shown). Finally, tumors with partial EMT were more frequently described in the N+ group than in the N0 group (n = 43, 60% vs n = 27, 38%, *P *<* *.01). Among the EMT effectors analyzed, *TWIST1* was the most frequently overexpressed EMT‐TF (n = 105, 73%), while *ZEB1* was the least overexpressed EMT‐TF (n = 9, 6%). *TWIST1* was more frequently overexpressed in the N+ group compared to the N0 group (n = 58, 81% vs n = 47, 66%, respectively, *P *=* *.04). *SNAI2* was also more frequently overexpressed in the N+ group compared to the N0 group (n = 47, 65% vs n = 29, 41%, respectively, *P *<* *.01).

**Table 2:** Expression of EMT markers, effectors, and inducers according to lymph node status

	All patients	N0	N+	*P* value
Total N (%)	143 (100)	71 (50)	72 (50)	
EMT markers^a^				
*CDH1* (E‐Cadherin)	132 (92)	67 (94)	65 (90)	NS
*CTNNB1* (β‐Catenin)	131 (92)	68 (96)	63 (88)	NS
*CDH2* (N‐Cadherin)	71 (50)	28 (39)	43 (60)	.01
*VIMENTIN*	18 (13)	5 (7)	13 (18)	.04
Partial EMT	70 (49)	27 (38)	43 (60)	<.01
EMT effectors^a^				
*SNAI1*	34 (24)	13 (18)	21 (29)	NS
*SNAI2*	76 (53)	29 (41)	47 (65)	<.01
*ZEB1*	9 (6)	2 (3)	7 (10)	NS
*TWIST1*	105 (73)	47 (66)	58 (81)	.04
*TWIST2*	32 (22)	12 (17)	20 (28)	NS
EMT inducers^a^				
TGFβ markers				
*TGFB1*	47 (33)	17 (24)	30 (42)	.02
*TGFBR1*	46 (32)	22 (31)	24 (33)	NS
*TGFBR2*	8 (6)	4 (6)	4 (6)	NS
c‐MET markers				
c‐MET IHC^b^	75 (52)	38 (47)	37 (46)	NS
*c‐MET*	75 (52)	36 (51)	39 (54)	NS
Hypoxia markers				
CAIX IHC^b^	15 (9)	7 (9)	8 (10)	NS
*HIF1*α	88 (62)	44 (62)	44 (61)	NS
*HIF2*α	1 (1)	1 (1)	0 (0)	NS

a Preserved expression for CDH1 and CTNNB1 genes, overexpression for the other genes.

b IHC: Immunohistochemistry scoring system for c‐MET and CAIX proteins: overexpression if ≥50% (intensity scores 2 + 3) of labeled tumors cells.

IHC performed on n = 160 tumors. EMT = Epithelial‐mesenchymal transition; N0 =  Patients with lymph node tumor status N0; N+ = Patients with lymph node tumor status N1 or N2; Partial EMT = overexpression of *CDH2* with normal expression of *CDH1*; CAIX = Carbonic anhydrase IX; NS = Not significant; *P*‐value <.05 statistically significant.

### 3.3 Expression of EMT inducers according to lymph node status

The *TGFB1* (TGFβ), *TGFBR1,* and *TGFBR2* genes were overexpressed in 47 (33%), 46 (32%), and 8 (6%) tumors, respectively (Table 2). *TGFB1* was more frequently overexpressed in the N+ group of tumors compared to the N0 group (n = 30, 42% vs n = 17, 24%, respectively, *P *=* *.02). C‐MET was shown to be overexpressed in 75 (52%) tumors by immunohistochemistry as well as RT‐qPCR analysis (Figure S2). CAIX protein was shown to be overexpressed in 15 (9%) tumors by immunohistochemistry. *HIF1*α and *HIF2*α overexpression were reported in 88 (62%) cases and 1 (1%) tumor, respectively. Neither c‐MET and CAIX protein nor *HIF1*α or *HIF2*α overexpression were associated with lymph node status.

### 3.4 Correlations between *SNAI2* and *TWIST1* expression and EMT markers according to lymph node status

As *SNAI2* was more frequently overexpressed in N+ compared to N0 tumors, we analyzed the EMT phenotype depending on *SNAI2* expression (Table 3). Epithelial markers were conserved in N+ tumors overexpressing *SNAI2,* suggesting a partial EMT phenotype. Indeed, mesenchymal markers were more often overexpressed when *SNAI2* expression was increased. *SNAI2* overexpression was significantly correlated with *CDH2* or *VIMENTIN* overexpression in the whole group, (*P *≤* *.001). Both correlations were observed in the N+ group (*P *=* *.003 and *P *=* *.002, respectively). In the N0 group, only *CDH2* overexpression was correlated with *SNAI2* overexpression (*P* = .001). *SNAI2* overexpression was significantly correlated with partial EMT features in the whole group (*P *<* *.001) as well as in the N0 or N+ groups (*P *=* *.003). In summary, *SNAI2* overexpression was correlated with partial EMT, which was more often observed in the N+ group and was particularly correlated with *CDH2* and *VIMENTIN* overexpression in the N+ group. As *TWIST1* was more frequently overexpressed in N+ tumors compared to N0 tumors, we also analyzed the EMT phenotype depending on *TWIST1* expression (Table 3). No correlation was observed between *TWIST1* overexpression and EMT epithelial marker expression, but mesenchymal markers were more often overexpressed when *TWIST1* expression was increased. Indeed, *TWIST1* overexpression was correlated with *VIMENTIN* overexpression in the N+ group of tumors (*P *=* *.05) and with *CDH2* overexpression in the N0 group of tumors (*P* < .001). Finally, *TWIST1* overexpression was associated with an increased number of tumors with a partial EMT phenotype, which were more frequent in the N+ group of tumors (n = 37, 64%) compared to the N0 group of tumors (n = 26, 55%).

**Table 3:** Correlations between *SNAI2 and TWIST1* expression and EMT markers according to lymph node status

	All patients			N0			N+		
Total N (%)	143 (100)			71 (50)			72 (50)		
*SNAI2* expression	Normal (n = 67)	Overexpressed (n = 76)	*P* value	Normal (n = 42)	Overexpressed (n = 29)	*P* value	Normal (n = 25)	Overexpressed (n = 47)	*P* value
*CDH1* (E‐Cadherin)									
Preserved	60 (90)	72 (95)	NS	40 (95)	27 (93)	NS	20 (80)	45 (96)	.05
Decreased	7 (10)	4 (5)		2 (5)	2 (7)		5 (20)	2 (4)	
*CTNNB1* (β‐Catenin)									
Preserved	60 (90)	71 (93)	NS	41 (98)	27 (93)	NS	19 (76)	44 (94)	.04
Decreased	7 (10)	5 (7)		1 (2)	2 (7)		6 (24)	3 (6)	
*CDH2* (N‐Cadherin)									
Normal	48 (72)	24 (32)	<.001	32 (76)	11 (38)	.001	16 (64)	13 (28)	.003
Overexpressed	19 (28)	52 (68)		10 (24)	18 (62)		9 (36)	34 (72)	
*VIMENTIN*									
Normal	66 (99)	59 (78)	<.001	41 (98)	25 (86)	NS	25 (100)	34 (72)	.002
Overexpressed	1 (1)	17 (22)		1 (2)	4 (14)		0 (0)	13 (28)	
Partial EMT									
No partial EMT	48 (72)	25 (33)	<.001	32 (76)	12 (41)	.003	16 (64)	13 (28)	.003
Partial EMT	19 (28)	51 (67)		10 (24)	17 (59)		9 (36)	34 (72)	

*TWIST1* expression	Normal (n = 38)	Overexpressed (n = 105)	*P* value	Normal (n = 24)	Overexpressed (n = 47)	*P* value	Normal (n = 14)	Overexpressed (n = 58)	*P* value
*CDH1* (E‐Cadherin)									
Preserved	34 (90)	98 (93)	NS	22 (92)	45 (96)	NS	12 (86)	53 (91)	NS
Decreased	4 (10)	7 (7)		2 (8)	2 (4)		2 (14)	5 (9)	
*CTNNB1* (β‐Catenin)									
Preserved	34 (90)	97 (92)	NS	23 (96)	45 (96)	NS	11 (79)	52 (90)	NS
Decreased	4 (10)	8 (8)		1 (4)	2 (4)		3 (21)	6 (10)	
*CDH2* (N‐Cadherin)									
Normal	31 (82)	41 (39)	<.001	23 (96)	20 (43)	<.001	8 (57)	21 (36)	NS
Overexpressed	7 (18)	64 (61)		1 (4)	27 (57)		6 (43)	37 (64)	
*VIMENTIN*									
Normal	36 (95)	89 (85)	NS	22 (92)	44 (94)	NS	14 (100)	45 (78)	.05
Overexpressed	2 (5)	16 (15)		2 (8)	3 (6)		0 (0)	13 (22)	
Partial EMT									
No partial EMT	31 (82)	42 (40)	<.001	23 (96)	21 (45)	<.001	8 (57)	21 (36)	NS
Partial EMT	7 (18)	63 (60)		1 (4)	26 (55)		6 (43)	37 (64)	

EMT = Epithelial‐mesenchymal transition; N0 = Patients with lymph node tumor status N0; N+ = Patients with lymph node tumor status N1 or N2; Partial EMT = overexpression of *CDH2* with normal expression of *CDH1;* NS = Not significant; *P*‐value <.05 statistically significant.

### 3.5 Correlations between *TGFB1*, c‐*MET*, and CAIX expression and EMT markers/effectors according to lymph node status

As *TGFB1* was more frequently overexpressed in N+ tumors compared to N0 tumors, we analyzed the population depending on the expression of *TGFB1*, one of the best inducers of EMT (Table 4). *TGFB1* overexpression was correlated with the overexpression of the mesenchymal markers *CDH2* and *VIMENTIN* (*P* < .001) and, consequently, with partial EMT (*P *<* *.001). In the N+ group of tumors, *TGFB1* overexpression was correlated with the expression of epithelial markers (*CDH1*,* P *=* *.02 and *CTNNB1*,* P *=* *.05), overexpression of mesenchymal markers (*CDH2*,* P *=* *.001 and *VIMENTIN*,* P *<* *.001) and, consequently, with partial EMT (*P *<* *.001). When *TGFB1* was overexpressed, partial EMT was more often reported in the N+ group (n = 25, 83%) than in the N0 group (n = 11, 65%). In the entire cohort, EMT‐TF overexpression (*SNAI1, SNAI2, ZEB1, TWIST1,* and *TWIST2*) was correlated with *TGFB1* overexpression. By subgroup analysis, EMT‐TF was differentially correlated with *TGFB1* overexpression, depending on the N status. Indeed, *ZEB1* overexpression alone was correlated with *TGFB1* overexpression only in the N+ group (*P* = .02). In summary, *TGFB1* overexpression was more frequently observed in the N+ group as well as correlated with partial EMT in the N0 group and especially in the N+ group. The correlation of *TGFB1* overexpression with EMT‐TF appeared to be different between the N0 and N+ groups, with *ZEB1* involvement in the latter.

**Table 4:** Correlations between *TGFB1*expression and EMT markers/effectors according to lymph node status

	All patients			N0			N+		
Total N (%)	143 (100)			71 (50)			72 (50)		
*TGFB1* expression	Normal (n = 96)	Overexpressed (n = 47)	*P* value	Normal (n = 54)	Overexpressed (n = 17)	*P* value	Normal (n = 42)	Overexpressed (n = 30)	*P* value
*CDH1* (E‐Cadherin)									
Preserved	85 (89)	47 (100)	.01	50 (93)	17 (100)	NS	35 (83)	30 (100)	.02
Decreased	11 (11)	0 (0)		4 (7)	0 (0)		7 (17)	0 (0)	
*CTNNB1* (β‐Catenin)									
Preserved	85 (89)	46 (98)	.05	51 (94)	17 (100)	NS	34 (81)	29 (97)	.05
Decreased	11 (11)	1 (2)		3 (6)	0 (0)		8 (19)	1 (3)	
*CDH2* (N‐Cadherin)									
Normal	61 (64)	11 (23)	<.001	37 (69)	6 (35)	.02	24 (57)	5 (17)	.001
Overexpressed	35 (36)	36 (77)		17 (31)	11 (65)		18 (43)	25 (83)	
*VIMENTIN*									
Normal	94 (98)	31 (66)	<.001	53 (98)	13 (76)	.01	41 (98)	18 (60)	< .001
Overexpressed	2 (2)	16 (34)		1 (2)	4 (24)		1 (2)	12 (40)	
Partial EMT									
No partial EMT	62 (65)	11 (23)	<.001	38 (70)	6 (35)	.01	24 (57)	5 (17)	< .001
Partial EMT	34 (35)	36 (77)		16 (30)	11 (65)		18 (43)	25 (83)	
*SNAI1*									
Normal	84 (88)	25 (53)	<.001	48 (89)	10 (59)	.01	36 (86)	15 (50)	.001
Overexpressed	12 (12)	22 (47)		6 (11)	7 (41)		6 (14)	15 (50)	
*SNAI2*									
Preserved	60 (63)	7 (15)	<.001	37 (69)	5 (29)	.005	23 (55)	2 (7)	<.001
Decreased	36 (37)	40 (85)		17 (31)	12 (71)		19 (45)	28 (93)	
*ZEB1*									
Normal	94 (98)	40 (85)	.006	53 (98)	16 (94)	NS	41 (98)	24 (80)	.02
Overexpressed	2 (2)	7 (15)		1 (2)	1 (6)		1 (2)	6 (20)	
*TWIST1*									
Normal	32 (33)	6 (13)	.006	21 (39)	3 (18)	NS	11 (26)	3 (10)	NS
Overexpressed	64 (67)	41 (87)		33 (61)	14 (82)		31 (74)	27 (90)	
*TWIST2*									
Normal	82 (85)	29 (62)	.002	48 (89)	11 (65)	.03	34 (81)	18 (60)	.05
Overexpressed	14 (18)	18 (38)		6 (11)	6 (35)		8 (19)	12 (40)	

EMT = Epithelial‐mesenchymal transition; N0 = Patients with lymph node tumor status N0; N+ = Patients with lymph node tumor status N1 or N2; Partial EMT = overexpression of *CDH2* with normal expression of *CDH1;* NS = Not significant; *P*‐value <.05 statistically significant.

We also analyzed two other potential inducers of EMT, c‐*MET,* and hypoxia (explored by CAIX protein expression) (Tables S1 and S2, Figure S2). C‐*MET* overexpression was correlated with EMT epithelial marker expression and overexpression of *VIMENTIN*, especially in the N+ group of tumors (*P *=* *.002). When c‐*MET* was overexpressed, partial EMT was more frequent in the N+ group (n = 25, 64%) compared to the N0 group of tumors (n = 16, 44%). No EMT‐TF was correlated with c‐*MET* overexpression in the N0 group of tumors, while *SNAI1* and *ZEB1* overexpression was correlated with c‐*MET* overexpression in the N+ group (*P* = .02 and *P* = .01, respectively). CAIX protein overexpression was correlated with overexpression of the mesenchymal marker *CDH2* only in the N+ group (*P* = .01). Moreover, all cases of N+ tumors overexpressing CAIX presented a partial EMT phenotype. No EMT‐TF was correlated with CAIX overexpression in the N0 group of tumors, while *SNAI2* and *ZEB1* overexpression was correlated with CAIX overexpression in the N+ group (*P* = .03). In summary, c‐*MET* and CAIX overexpression was associated with mesenchymal markers more often in the N+ group. Finally, some EMT‐TFs were correlated with overexpression of the EMT inducers c‐*MET* or CAIX only in the N+ group of tumors.

### 3.6 Survival analysis

**Figure 1:**
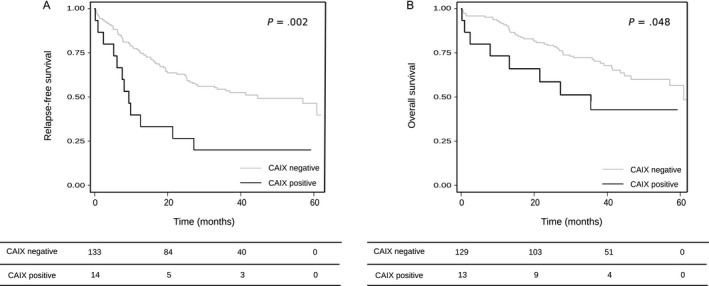
Relapse‐free survival (A) and Overall survival (B) and CAIX expression. Median time of clinical follow‐up: 37 mo (range 1‐63 mo). Median RFS time: 9 mo (CAIX positive) vs 42 mo (CAIX negative), *P *=* *.002. Median OS time: 27 mo (CAIX positive) vs not reached (CAIX negative), *P *=* *.048. CAIX: Carbonic anhydrase IX. Immunohistochemistry scoring system for CAIX protein: negative if <50% (intensity scores 2 + 3) of labeled tumor cells, positive if ≥50% (intensity scores 2 + 3) of labeled tumors cells

The median time of clinical follow‐up of patients was 37 months (range 1‐63 months). In univariate analysis, neither EMT markers nor EMT effectors were observed as prognostic factors in the present study (Table 5) (Table S3). The EMT inducer CAIX protein was a bad prognostic factor for RFS and OS (*P *=* *.003 and *P *=* *.05, respectively). Indeed, there was a significant decrease in RFS for patients with tumors overexpressing CAIX (median RFS time is 9 months vs 42 months, *P *=* *.002), and the same results were observed for OS (median OS time is 27 months vs not reached, *P *=* *.048) (Figure 1). In multivariate analyses, CAIX was an independent prognostic factor (HR 2.99, 95% CI: 1.58‐5.65, *P *=* *.001 for RFS and HR 2.38, 95% CI: 1.10‐5.14, *P *=* *.03 for OS).

**Table 5:** Multivariate Cox model analysis for relapse‐free survival and overall survival

All patients (N = 160)	Relapse‐free survival			Overall survival		
	HR	95% CI	*P* value	HR	95% CI	*P* value
Gender: female vs male	1.10	0.66‐1.82	.72	1.03	0.56‐1.89	.92
Age at diagnosis: >60 vs ≤60 y	1.11	0.69‐1.81	.65	1.03	0.58‐1.81	.91
UICC stage: I‐II vs III	2.20	1.37‐3.53	.001	1.62	0.92‐2.86	.10
Neoadjuvant chemotherapy: yes vs no	1.88	1.02‐3.45	.04	2.09	1.06‐4.12	.03
IHC^a^ CAIX positive vs negative	2.99	1.58‐5.65	.001	2.38	1.10‐5.14	.03

a IHC: Immunohistochemistry scoring system for CAIX protein, positive if ≥50% (intensity scores 2 + 3) of labeled tumors cells, negative if <50% (intensity scores 2 + 3) of labeled tumor cells.

HR, Hazard Ratio; CI, Confidence Interval; *P*‐value <.05 statistically significant.

## 4 DISCUSSION

Epithelial to mesenchymal transition may be involved in cancer progression and in lymph node progression of NSCLC. Previous studies have been conducted to assess individual EMT markers in NSCLC, but to our knowledge, no studies have examined several EMT markers simultaneously. In the present study, we investigated the expression of EMT markers, effectors, and inducers in early stage NSCLC and separated tumors into N0 and N+ groups to describe their combined role in the development of lymph node metastasis. E‐Cadherin and β‐Catenin are classical markers for the epithelial phenotype. The present results showed that the majority of tumors had conserved expression of the epithelial markers *CDH1* (92%) and *CTNNB1* (92%). Previous studies on E‐Cadherin expression in NSCLC tested by immunohistochemistry report conserved protein expression ranging from 32% to 88% in tumors depending on the IHC cutoff value.^22^, ^23^, ^24^, ^25^, ^26^, ^27^, ^28^, ^29^ Moreover, an association of E‐Cadherin loss of IHC expression and the N+ status of patients was reported in several stages I to IV NSCLC cohorts.^23^, ^24^, ^28^, ^29^, ^30^, ^31^, ^32^, ^33^ Two other studies that analyzed *CDH1* expression in stage I to III NSCLC tumors reported an opposite association between *CDH1* expression and N status, with one study reporting preserved *CDH1* expression and the other study reporting lost *CDH1* expression.^34^, ^35^ In summary, loss of epithelial markers, such as *CDH1* and *CTNNB1*, was a rare event in the present early stage NSCLC cohort.

Vimentin is a key marker of the mesenchymal phenotype.^9^ N‐Cadherin is less used, but it is interesting to analyze its expression combined with E‐Cadherin to follow the “Cadherin switch” of EMT.^9^ The present results show overexpression of *CDH2* and *VIMENTIN* in 50% and 13% of tumors, respectively, with a correlation with N+ status. Expression of N‐Cadherin in NSCLC has been studied by IHC in several NSCLC cohorts, with 9% to 43% of tumors showing overexpression.^22^, ^36^, ^37^, ^38^ IHC expression of VIMENTIN was also previously investigated in NSCLC, with 7%‐66% of overexpression depending on the IHC cutoff value.^16^, ^24^, ^27^, ^29^, ^30^, ^38^, ^39^, ^40^, ^41^, ^42^, ^43^, ^44^, ^45^ One study quantified *CDH2* expression by RT‐qPCR in 30 NSCLC (stages I to IV) and found that 67% of tumors had N‐Cadherin overexpression, with a bad prognosis value.^37^ Four studies have found an association between VIMENTIN IHC overexpression and N+ status of NSCLC.^24^, ^29^, ^30^, ^45^ VIMENTIN overexpression was reported to be a bad prognostic marker in some NSCLC cohorts.^24^, ^27^, ^29^, ^39^, ^43^, ^44^, ^45^ In summary, the present results suggest a role for *CHD2* and *VIMENTIN* in the development of lymph node metastasis, even if they are not prognostic factors in early stages of NSCLC.

Total EMT (loss of *CDH1* expression associated with overexpression of *CDH2*) was observed in only one tumor in the present study. Partial EMT (expression of *CDH1* associated with overexpression of *CDH2*) was observed in 49% of tumors. This partial EMT was significantly more frequent in the N+ group of patients, suggesting a role for EMT in lymph node progression in early stage lung cancers. In fact, some authors have recently proposed that tumors with a partial EMT phenotype (defined as expressing of epithelial and mesenchymal markers) could have more metastatic features than tumors with a total EMT phenotype.^46^, ^47^ This finding could be explained through the migration of tumoral cell clusters instead of isolated tumor cells.

Among EMT inducers, TGFβ is a major EMT inducer in cancer and induces the EMT‐TFs SNAI1, SNAI2, ZEB1, and ZEB2.^18^ We showed that *TGFB1* is overexpressed in 33% of tumors, with a greater frequency observed in the N+ group of tumors. In previous studies, TGFβ protein or gene expression analyses have revealed a large range of overexpression, from 18% to 87%, in NSCLC depending on the technique (protein or gene analysis) or cutoff.^39^, ^48^, ^49^, ^50^ The present study is, to our knowledge, the first to identify a correlation between *TGFB1* overexpression and N+ status in NSCLC patients. We also showed that *TGFB1* overexpression is correlated with overexpression of the mesenchymal markers *CDH2* and *VIMENTIN* and partial EMT, especially in the N+ group of patients. One study found that the TGFβ‐activated SMAD3/4 complex upregulates N‐Cadherin expression in NSCLC cells and that SMAD3/4 expression was correlated with N‐Cadherin expression in a NSCLC cohort.^51^ Another in vitro assay showed that TGFβ could enhance *VIMENTIN* and *SNAI2* expression in NSCLC cells.^52^ In the present study, we found correlations between the overexpression of *TGB1* and EMT‐TFs *SNAI1, SNAI2, ZEB1, TWIST1,* and *TWIST2*, but with differences among N status. Indeed, *ZEB1* overexpression alone was correlated with overexpressed *TGFB1*, but only in the N+ group. In summary, TGFβ may be a strong EMT inducer in the present cohort, especially in the N+ group, suggesting its role in lymph node progression in early stages of NSCLC patients.

Another EMT inducer, c‐*MET*, was overexpressed in half of the tumors (52%) examined in the present study, which corresponds to the frequency of other studies in NSCLC, but without differences between the N+ and N0 groups.^21^, ^53^, ^54^ In the present study, overexpression of *c‐MET* was mostly correlated with *VIMENTIN* overexpression. No correlation was observed with *CDH2* overexpression, independent of the group of patients. We also described a correlation between *c‐MET* and EMT‐TFs *SNAI1* and *ZEB1* overexpression, but only in the N+ group. Some authors suggest that SNAI1 could be induced by c‐MET, and a recent study on hepatocellular carcinoma cells and tumors showed a correlation of c‐MET overexpression with VIMENTIN, SNAI1 and ZEB1 overexpression that could be explained by overexpression of the transcriptional factor FoxM1.^55^, ^56^, ^57^ In summary, c‐*MET* may be a less important EMT inducer than *TGFB1* in NSCLC patients, without differences among the N status, but involving different EMT‐TFs, such as *ZEB1* and *SNAI1*, only in the N+ group of tumors.

Hypoxia is also an EMT inducer associated with the TGFβ and c‐MET pathways.^55^, ^58^ Overexpression of *HIF1*α and *HIF2*α was observed in 63% and 1% of tumors in the present cohort, respectively, which is in the range of different studies for HIF1α but under the range for HIF2α.^59^, ^60^, ^61^ As CAIX is a target protein of *HIF1*α, but more stable than HIF1α, we examined CAIX protein expression with EMT features. CAIX was overexpressed in only 9% of the tumors examined in the present study, which is lower than that reported in other studies on NSCLC cohorts, in which 24% to 38% of tumors showed overexpressed CAIX protein.^60^, ^62^, ^63^ In the present study, when CAIX overexpression was not different between N groups, its correlation with the mesenchymal marker *CDH2* and partial EMT was only observed in the N+ group. CAIX overexpression was also correlated with EMT‐TFs *SNAI2* and *ZEB1* overexpression only in the N+ group. No previous studies have reported a correlation between CAIX and EMT markers/effectors in NSCLC. However, in tongue squamous cell carcinoma (in vitro and in tumor tissue samples), an investigation showed that CAIX and ZEB1 protein expression are correlated and that CAIX  ould be regulated by ZEB1.^64^ A correlation between CAIX and SNAI2 was also reported by IHC analysis in basal‐like breast carcinomas.^65^ In summary, CAIX may, such as c‐*MET*, be a less important EMT inducer than *TGFB1* in NSCLC patients, without differences depending on the N status, but involving different EMT‐TFs, only in the N+ group of tumors.

Two EMT effectors, *SNAI2* and *TWIST1*, were more often overexpressed in the N+ group of tumors. In the present study, *SNAI2* was overexpressed in 53% of cases, and in the N+ group, *SNAI2* was more frequently overexpressed and primarily correlated with the mesenchymal markers and EMT inducers *TGFB1* and CAIX. The present study is, to our knowledge, the first to report these associations in a NSCLC cohort. Nevertheless, the association between SNAI2 overexpression and the presence of lymph node metastasis has already been described by protein and gene analysis in tongue squamous cell carcinoma and breast and colorectal cancers, as well as the correlation of SNAI2 overexpression with N‐Cadherin and Vimentin overexpression.^66^, ^67^, ^68^ *TWIST1* was overexpressed in 73% of the present cases and more frequently overexpressed in the N+ group. TWIST1 was investigated in several NSCLC cohorts by IHC, with overexpression ranging from 11% to 68% 37, 38, 43, 69, 70, 71 or gene expression at 57%.^37^ Similar to the present study, a correlation between TWIST and CDH2 protein expression was found in a NSCLC cohort, but without data regarding the N status.^38^ As in the present study, a previous study found an association of TWIST1 protein overexpression with lymph node metastasis in lung cancer, supporting the role of TWIST1 in local progression.^72^ In the present work, *TWIST1* overexpression was associated with *VIMENTIN* overexpression, especially in N+ tumors. The present study showed that even if *TWIST1* was related to partial EMT phenotype, its overexpression was not correlated with the overexpression of EMT inducers, such as *TGFB1*,* c‐MET,* and CAIX, in the N+ group of tumors. These results suggest that *TWIST1* could be involved in the EMT process by other inducers, such as EGFR.^73^ Finally, even if *ZEB1* is not differentially overexpressed between the N+ and N0 groups, it appears to be correlated with the three EMT inducers *TGFB1*,* c‐MET,* and *CAIX* only in the N+ group of tumors. In summary, the present results suggest that EMT‐TFs are differentially related to EMT inducers in the N+ group of tumors and could promote the development of lymph node metastasis in early stage NSCLC.

Finally, the present results showed no prognostic value of EMT markers or effectors in the early stages of NSCLC. Nevertheless, the EMT inducer CAIX was shown to be an independent bad prognosis factor for RFS as well as for OS. The prognostic value of CAIX has also been reported in studies of NSCLC.^60^, ^62^, ^74^, ^75^ HIF1α and HIF2α protein overexpression was shown to have a bad prognostic value in NSCLC, and one meta‐analysis based on 30 studies also showed an association between HIF1α overexpression and lymph node‐positive tumors in NSCLC.^59^, ^61^, ^62^, ^74^, ^76^ Finally, CAIX overexpression had a negative impact on prognosis in the present cohort of early stage NSCLC patients. This effect could be explained by the potential role of CAIX in the development of a partial EMT phenotype in the N+ group through the EMT effectors *SNAI1* and *ZEB1*.

In conclusion, although the present study is retrospective with a small number of NSCLC patients for whom frozen tumors were available, we showed for the first time that the EMT‐TFs *SNAI2* and *TWIST1* (and to a lesser extent *ZEB1*) could be involved through different pathways in lymph node progression. Only EMT inducer CAIX overexpression was a bad prognosis factor. In the future, these findings could be validated by further “in vitro*”* models as well as prospective clinical studies.

## CONFLICT OF INTEREST

The authors declare no conflicts of interest.
