# Supplementary figures and images for: SNAI2 and TWIST1 in lymph node progression in early stages of NSCLC patients
Source: Cancer Med. 2018 May 29;7(7):3278–91. doi: 10.1002/cam4.1545 (PMC6051239; doi:10.1002/cam4.1545)

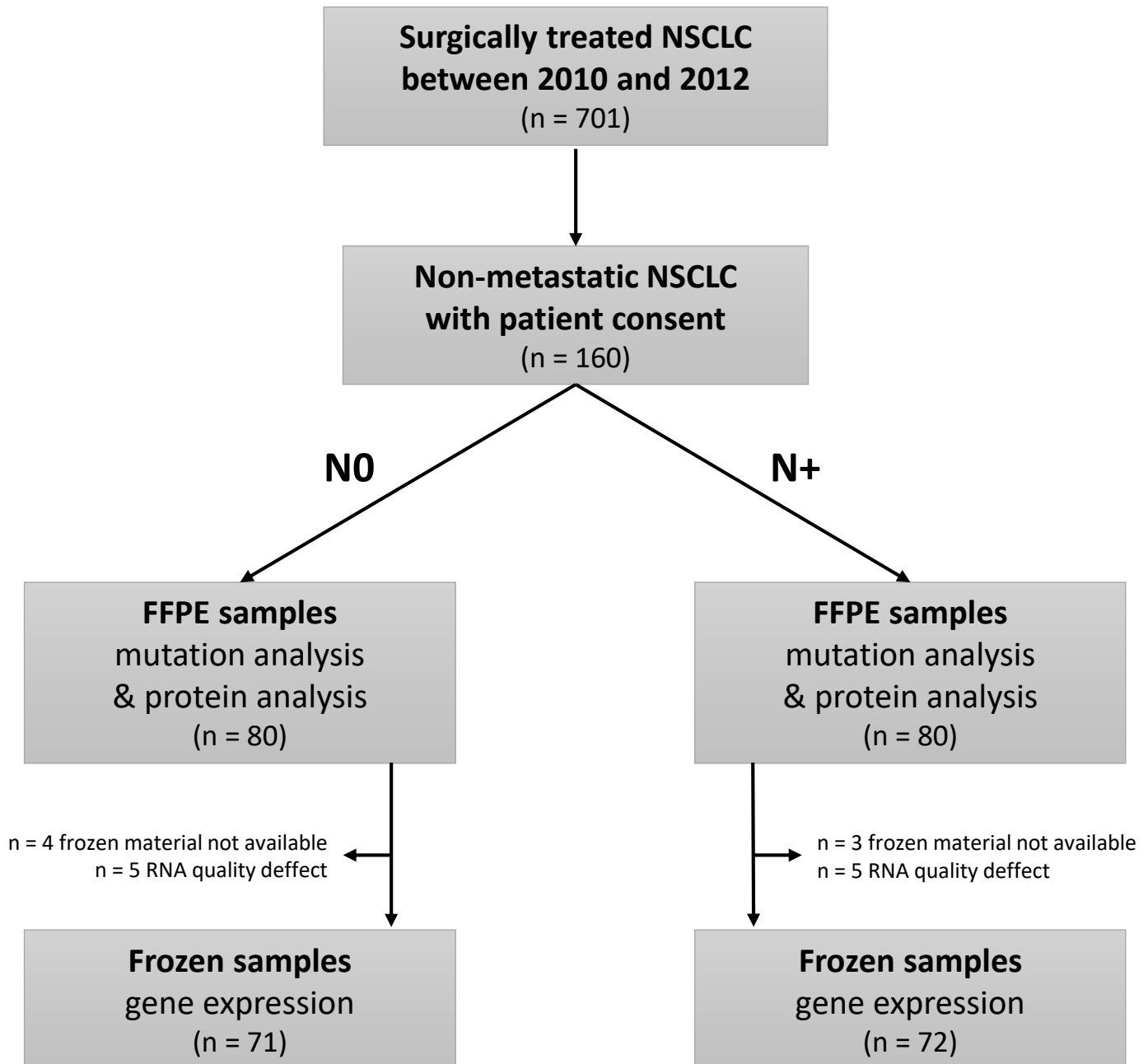

Supplementary data, Figure S1.

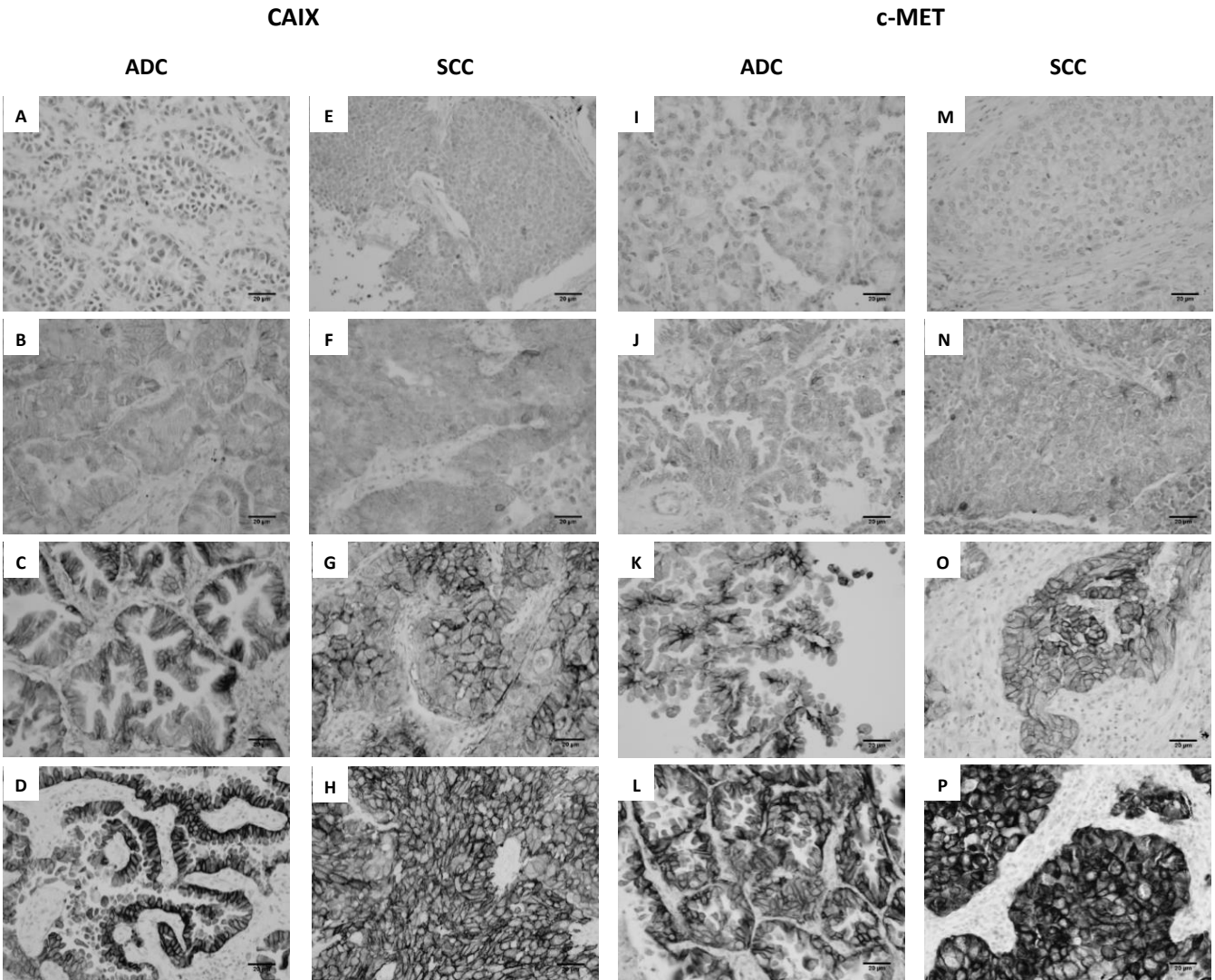

Supplementary data, Figure S2.

Supplement: Supplementary file 1 [file CAM4-7-3278-s001.pdf]
