# Supplementary material for: SNAI2 and TWIST1 in lymph node progression in early stages of NSCLC patients
Source: Cancer Med. 2018 May 29;7(7):3278–91. doi: 10.1002/cam4.1545 (PMC6051239; doi:10.1002/cam4.1545)
Supplement: Supplementary file 2 [file CAM4-7-3278-s002.docx]

TABLES

| **Table S1. Correlations between *c-MET* expression and EMT markers / effectors according to lymph node status** | | | | | | | | | |
| --- | --- | --- | --- | --- | --- | --- | --- | --- | --- |
|  | **All patients** | | | **N0** | | | **N+** | | |
| **Total N (%)** | 143 (100) | | | 71 (50) | | | 72 (50) | | |
| ***c-MET* expression** | **normal (n = 68)** | **Over-expressed (n = 75)** | ***P* value** | **normal (n = 35)** | **Over-expressed (n = 36)** | ***P* value** | **normal (n = 33)** | **Over-expressed (n = 39)** | ***P* value** |
| ***CDH1* (E-Cadherin)** |  | | 0,003 |  | | NS |  | | 0,003 |
| Preserved | 58 (85) | 74 (99) |  | 32 (91) | 35 (97) |  | 26 (79) | 39 (100) |  |
| Decreased | 10 (15) | 1 (1) |  | 3 (9) | 1 (3) |  | 7 (21) | 0 (0) |  |
| ***CTNNB1* (β-Catenin)** |  | | 0,01 |  | | NS |  | | 0,04 |
| Preserved | 58 (85) | 73 (97) |  | 32 (91) | 36 (100) |  | 26 (79) | 37 (95) |  |
| Decreased | 10 (15) | 2 (3) |  | 3 (9) | 0 (0) |  | 7 (21) | 2 (5) |  |
| ***CDH2* (N-Cadherin)** |  | | NS |  | | NS |  | | NS |
| Normal | 38 (56) | 34 (45) |  | 23 (66) | 20 (56) |  | 15 (45) | 14 (36) |  |
| Overexpressed | 30 (44) | 41 (55) |  | 12 (34) | 16 (44) |  | 18 (55) | 25 (64) |  |
| ***VIMENTIN*** |  | | < 0,001 |  | | 0,03 |  | | 0,002 |
| Normal | 67 (99) | 58 (77) |  | 35 (100) | 31 (86) |  | 32 (97) | 27 (69) |  |
| Overexpressed | 1 (1) | 17 (23) |  | 0 (0) | 5 (14) |  | 1 (3) | 12 (31) |  |
| **Partial EMT** |  | | NS |  | | NS |  | | NS |
| No partial EMT | 39 (57) | 34 (45) |  | 24 (69) | 20 (56) |  | 15 (45) | 14 (36) |  |
| Partial EMT | 29 (43) | 41 (55) |  | 11 (31) | 16 (44) |  | 18 (55) | 25 (64) |  |
| ***SNAI1*** |  | | 0,004 |  | | NS |  | | 0,02 |
| Normal | 59 (87) | 50 (67) |  | 31 (89) | 27 (75) |  | 28 (85) | 23 (59) |  |
| Overexpressed | 9 (13) | 25 (33) |  | 4 (11) | 9 (25) |  | 5 (15) | 16 (41) |  |
| ***SNAI2*** |  | | NS |  | | NS |  | | NS |
| Preserved | 35 (51) | 32 (43) |  | 22 (63) | 20 (56) |  | 13 (39) | 12 (31) |  |
| Decreased | 33 (49) | 43 (57) |  | 13 (37) | 16 (44) |  | 20 (61) | 27 (69) |  |
| ***ZEB1*** |  | | 0,002 |  | | NS |  | | 0,01 |
| Normal | 68 (100) | 66 (88) |  | 35 (100) | 34 (94) |  | 33 (100) | 32 (82) |  |
| Overexpressed | 0 (0) | 9 (12) |  | 0 (0) | 2 (6) |  | 0 (0) | 7 (18) |  |
| ***TWIST1*** |  | | NS |  | | NS |  | | NS |
| Normal | 20 (29) | 18 (24) |  | 12 (34) | 12 (33) |  | 8 (24) | 6 (15) |  |
| Overexpressed | 48 (71) | 57 (76) |  | 23 (66) | 24 (67) |  | 25 (76) | 33 (85) |  |
| ***TWIST2*** |  | | NS |  | | NS |  | | NS |
| Normal | 56 (82) | 55 (73) |  | 30 (86) | 29 (81) |  | 26 (79) | 26 (67) |  |
| Overexpressed | 12 (18) | 20 (27) |  | 5 (14) | 7 (19) |  | 7 (21) | 13 (33) |  |
| EMT = Epithelial-mesenchymal transition; N0 = Patients with lymph node tumor status N0; N+ = Patients with lymph node tumor status N1 or N2; Partial EMT = overexpression of *CDH2* with normal expression of *CDH1;* NS = Not significant; *P*-value < 0,05 statistically significant. | | | | | | | | | |

| **Table S2. Correlations between CAIX expression and EMT markers / effectors according to lymph node status** | | | | | | | | | |
| --- | --- | --- | --- | --- | --- | --- | --- | --- | --- |
|  | **All patients** | | | **N0** | | | **N+** | | |
| **Total N (%)** | 143 (100) | | | 71 (50) | | | 72 (50) | | |
| **CAIX IHC† expression** | **negative (n = 128)** | **positive (n = 15)** | ***P* value** | **negative (n = 64)** | **positive (n = 7)** | ***P* value** | **negative (n = 64)** | **positive (n = 8)** | ***P* value** |
| ***CDH1* (E-Cadherin)** |  | | NS |  | | NS |  | | NS |
| Preserved | 118 (92) | 14 (93) |  | 61 (95) | 6 (86) |  | 57 (89) | 8 (100) |  |
| Decreased | 10 (8) | 1 (7) |  | 3 (5) | 1 (14) |  | 7 (11) | 0 (0) |  |
| ***CTNNB1* (β-Catenin)** |  | | NS |  | | NS |  | | NS |
| Preserved | 117 (91) | 14 (93) |  | 62 (97) | 6 (86) |  | 55 (86) | 8 (100) |  |
| Decreased | 11 (9) | 1 (7) |  | 2 (3) | 1 (14) |  | 9 (14) | 0 (0) |  |
| ***CDH2* (N-Cadherin)** |  | | 0,05 |  | | NS |  | | 0,01 |
| Normal | 68 (53) | 4 (27) |  | 39 (61) | 4 (57) |  | 29 (43) | 0 (0) |  |
| Overexpressed | 60 (47) | 11 (73) |  | 25 (39) | 3 (43) |  | 35 (57) | 8 (100) |  |
| ***VIMENTIN*** |  | | NS |  | | NS |  | | NS |
| Normal | 112 (88) | 13 (87) |  | 59 (92) | 7 (100) |  | 53 (83) | 6 (75) |  |
| Overexpressed | 16 (12) | 2 (13) |  | 5 (8) | 0 (0) |  | 11 (17) | 2 (25) |  |
| **Partial EMT** |  | | 0,04 |  | | NS |  | | 0,01 |
| No partial EMT | 69 (54) | 4 (27) |  | 40 (63) | 4 (57) |  | 29 (43) | 0 (0) |  |
| Partial EMT | 59 (46) | 11 (73) |  | 24 (37) | 3 (43) |  | 35 (57) | 8 (100) |  |
| ***SNAI1*** |  | | NS |  | | NS |  | | NS |
| Normal | 99 (77) | 10 (67) |  | 52 (81) | 6 (86) |  | 47 (73) | 4 (50) |  |
| Overexpressed | 29 (33) | 5 (33) |  | 12 (19) | 1 (14) |  | 17 (27) | 4 (50) |  |
| ***SNAI2*** |  | | 0,02 |  | | NS |  | | 0,03 |
| Preserved | 64 (50) | 3 (20) |  | 39 (61) | 3 (43) |  | 25 (39) | 0 (0) |  |
| Decreased | 64 (50) | 12 (80) |  | 25 (39) | 4 (57) |  | 39 (61) | 8 (100) |  |
| ***ZEB1*** |  | | 0,05 |  | | NS |  | | 0,03 |
| Normal | 122 (95) | 12 (80) |  | 62 (97) | 7 (100) |  | 60 (94) | 5 (63) |  |
| Overexpressed | 6 (5) | 3 (20) |  | 2 (3) | 0 (0) |  | 4 (6) | 3 (37) |  |
| ***TWIST1*** |  | | NS |  | | NS |  | | NS |
| Normal | 34 (27) | 4 (27) |  | 21 (33) | 3 (43) |  | 13 (20) | 1 (12) |  |
| Overexpressed | 94 (73) | 11 (73) |  | 43 (67) | 4 (57) |  | 51 (80) | 7 (88) |  |
| ***TWIST2*** |  | | NS |  | | NS |  | | NS |
| Normal | 99 (77) | 12 (80) |  | 53 (83) | 6 (86) |  | 46 (72) | 6 (75) |  |
| Overexpressed | 29 (23) | 3 (20) |  | 11 (17) | 1 (14) |  | 18 (28) | 2 (25) |  |
| † IHC: Immunohistochemistry scoring system for CAIX protein: negative if < 50% (intensity scores 2+3) of labelled tumor cells, positive if ≥ 50% (intensity scores 2+3) of labelled tumors cells. EMT = Epithelial-mesenchymal transition; N0 = Patients with lymph node tumor status N0; N+ = Patients with lymph node tumor status N1 or N2; Partial EMT = overexpression of *CDH2* with normal expression of *CDH1;* NS = Not significant; *P*-value < 0,05 statistically significant. | | | | | | | | | |

| **Table S3. Univariate Cox Model Analysis for Relapse-free survival and Overall survival** | | | | | | |
| --- | --- | --- | --- | --- | --- | --- |
|  | **Relapse-free survival** | | | **Overall survival** | | |
| **All patients (N = 160)** | **HR** | **95% CI** | ***P* value** | **HR** | **95% CI** | ***P* value** |
| Gender: female vs. male | 1,07 | 0,67 - 1,70 | 0,79 | 1,03 | 0,59 - 1,81 | 0,92 |
| Age at diagnosis: > 60 vs. ≤ 60 years | 0,99 | 0,64 - 1,54 | 0,96 | 0,89 | 0,53 - 1,50 | 0,66 |
| Smocking history: ever vs. never | 1,51 | 0,69 - 3,27 | 0,30 | 1,72 | 0,62 - 4,76 | 0,30 |
| Neoadjuvant chemotherapy: yes vs. no | 2,39 | 1,38 - 4,15 | 0,002 | 2,41 | 1,32 - 4,42 | 0,004 |
| TKI treatment: yes vs. no | 1,06 | 1,69 - 4,91 | < 0,001 | 0,62 | 0,94 - 3,67 | 0,08 |
| Histology: adenocarcinoma vs. others† | 0,80 | 0,50 - 1,28 | 0,36 | 0,61 | 0,35 - 1,05 | 0,07 |
| T-stage: T3-T4 vs. T1-T2 | 1,47 | 0,93 - 2,30 | 0,10 | 1,60 | 0,94 - 2,73 | 0,09 |
| UICC stage: I-II vs. III | 2,34 | 1,51 - 3,63 | < 0,001 | 1,91 | 1,14 - 3,24 | 0,01 |
| Any mutation: yes vs. no | 0,92 | 0,60 - 1,42 | 0,72 | 0,66 | 0,38 - 1,12 | 0,12 |
| *CDH1* (E-Cadherin): preserved vs. decreased | 0,63 | 0,30 - 1,31 | 0,22 | 0,48 | 0,21 - 1,06 | 0,07 |
| *CTNNB1* (β-catenin): preserved vs. decreased | 0,86 | 0,37 - 1,98 | 0,72 | 0,53 | 0,23 - 1,24 | 0,14 |
| *CDH2* (N-Cadherin): overexpressed vs. normal | 0,88 | 0,55 - 1,40 | 0,58 | 1,15 | 0,66 - 2,00 | 0,64 |
| *VIMENTIN*: overexpressed vs. normal | 0,45 | 0,18 - 1,12 | 0,09 | 0,7 | 0,28 - 1,75 | 0,44 |
| partial EMT: yes vs. no | 0,85 | 0,53 - 1,35 | 0,49 | 1,09 | 0,62 - 1,89 | 0,77 |
| *SNAI1*: overexpressed vs. normal | 0,98 | 0,56 - 1,71 | 0,94 | 1,15 | 0,61 - 2,16 | 0,67 |
| *SNAI2*: overexpressed vs. normal | 1,10 | 0,69 - 1,76 | 0,68 | 1,15 | 0,83 - 2,58 | 0,19 |
| *ZEB1*: overexpressed vs. normal | 1,03 | 0,37 - 2,85 | 0,95 | 0,96 | 0,30 - 3,09 | 0,94 |
| *TWIST1*: overexpressed vs. normal | 1,18 | 0,69 - 2,00 | 0,55 | 1,48 | 0,74 - 2,96 | 0,27 |
| *TWIST2*: overexpressed vs. normal | 0,95 | 0,53 - 1,70 | 0,85 | 0,79 | 0,38 - 1,63 | 0,52 |
| *TGFB1*: overexpressed vs. normal | 0,68 | 0,40 - 1,18 | 0,17 | 1,01 | 0,56 - 1,83 | 0,97 |
| *TGFBR1*: overexpressed vs. normal | 0,69 | 0,40 - 1,18 | 0,17 | 0,86 | 0,47 - 1,58 | 0,64 |
| *TGFBR2*: overexpressed vs. normal | 0,65 | 0,20 - 2,06 | 0,46 | 0,97 | 0,30 - 3,11 | 0,95 |
| IHC^‡^ c-MET: positive vs. negative | 0,83 | 0,54 - 1,28 | 0,40 | 0,6 | 0,36 - 1,02 | 0,06 |
| *c-MET*: overexpressed vs. normal | 0,78 | 0,49 - 1,24 | 0,29 | 0,85 | 0,49 - 1,48 | 0,56 |
| IHC^‡^ CAIX: positive vs. negative | 2,58 | 1,39 - 4,78 | 0,003 | 2,10 | 0,99 - 4,43 | 0,05 |
| *HIF1α*: overexpressed vs. normal | 0,76 | 0,48 - 1,21 | 0,25 | 1,04 | 0,58 - 1,85 | 0,09 |
| *HIF2α*: overexpressed vs. normal | 0,05 | 0 - 3189,20 | 0,59 | 0,05 | 0 - 34207,50 | 0,66 |
| †Squamous cell carcinomas, adenosquamous carcinomas and carcinomas with neuroendocrine features. ^‡^ IHC: Immunohistochemistry scoring system for c-MET and CAIX proteins, positive if ≥ 50% (intensity scores 2+3) of labelled tumors cells, negative if < 50% (intensity scores 2+3) of labelled tumor cells. HR = Hazard Ratio; CI = Confidence Interval; EMT= Epithelial-mesenchymal transition; Any mutation = EGFR, KRAS, BRAF, HER2, PI3KCA mutation or ALK rearrangement; Partial EMT= overexpression of *CDH2* with normal expression of *CDH1;* TKI = EGFR Tyrosine-kinase inhibitor; *P*-value < 0,05 statistically significant. | | | | | | |

TITLE AND LEGEND TO FIGURES

**Figure S1. Flow -chart**

NSCLC: Non-Small-Cell Lung Carcinoma; N0 = Patients with lymph node tumor status N0; N+ = Patients with lymph node tumor status N1 or N2; FFPE : formalin fixed, paraffin embedded material; Mutation status: *EGFR, KRAS, HER2, BRAF, PIK3CA* mutation status and *ALK* rearrangement; IHC: Immunochemistry analysis for c-MET and CAIX; Frozen material not available if necrosis or depleted; RNA quality defect: RNA Integrity Number < 5; Gene expression: by RT-qPCR for *CDH1*, *CDH2*, *CTNNB1*, *VIMENTIN*, *SNAI1*, *SNAI2*, *ZEB1*, *TWIST1*, *TWIST2*, *c-MET*, *TGFB1*, *TGFBR1*, *TGFBR2*, *HIF1α* and *HIF2α*.

**Figure S2. CAIX and c-MET expression by immunohistochemistry**

CAIX expression in adenocarcinoma (**ADC**) and squamous cell carcinoma (**SCC**). Staining levels: negative (**A**, **E**), 1+ (**B**, **F**), 2+ (**C**, **G**) and 3+ (**D**, **H**). C**-**MET expression in adenocarcinoma (**ADC**) and squamous cell carcinoma (**SCC**). Staining levels: negative (**I**, **M**), 1+ (**J**, **N**), 2+ (**K**, **O**) and 3+ (**L**, **P**). Scale bars represent 20µm.
